# Exploring the Components, Asymmetry and Distribution of Relationship Quality in Wild Barbary Macaques (*Macaca sylvanus*)

**DOI:** 10.1371/journal.pone.0028826

**Published:** 2011-12-14

**Authors:** Richard McFarland, Bonaventura Majolo

**Affiliations:** School of Psychology, University of Lincoln, Lincoln, United Kingdom; Cajal Institute, Consejo Superior de Investigaciones Científicas, Spain

## Abstract

Social relationships between group members are a key feature of many animal societies. The quality of social relationships has been described by three main components: value, compatibility and security, based on the benefits, tenure and stability of social exchanges. We aimed to analyse whether this three component structure could be used to describe the quality of social relationships in wild Barbary macaques (*Macaca sylvanus*). Moreover, we examined whether relationship quality was affected by the sex, age and rank differences between social partners, and investigated the asymmetric nature of social relationships. We collected over 1,900 hours of focal data on seven behavioural variables measuring relationship quality, and used principal component analysis to investigate how these variables clustered together. We found that relationship quality in wild Barbary macaques can be described by a three component structure that represents the value, compatibility and security of a relationship. Female-female dyads had more valuable relationships and same-age dyads more compatible relationships than any other dyad. Rank difference had no effect on the quality of a social relationship. Finally, we found a high degree of asymmetry in how members of a dyad exchange social behaviour. We argue that the asymmetry of social relationships should be taken into account when exploring the pattern and function of social behaviour in animal societies.

## Introduction

In animal societies, social bonds between group members provide fitness benefits such as improved access to food and mating opportunities, protection from predators, and reduced infanticide risk [1]–[4]. In primates (including humans), high quality, friendly social relationships are also considered to have a positive impact on an individual's reproductive success [5]–[8]. For example, individuals that are more integrated into their social group (measured by the quality of their social relationships with other group members) experience higher rates of infant survival [6], [7] and tend to sire more offspring [8]. Moreover, relationship quality can modulate conflict resolution [9]–[11] or the reciprocal exchanges of social resources [12]–[14]. Therefore, investigating the quality and distribution of social relationships in animal and human societies is crucial to understand group processes and social evolution.

Kummer [15] and Hinde [16] were the first to identify that group-living animals can establish long-term social relationships with their group companions and that these relationships can be described by the frequency and type of behaviour exchanged between two social partners. Following this approach, Cords and Aureli [9] suggested a theoretical framework whereby the quality of social relationships can be described by three components: value, compatibility and security. The value of a social relationship encapsulates the different benefits that an individual gains from their social relationship. In non-human primates, for example, these benefits might include grooming and tolerance around food, as these behaviours contribute to the fitness of an individual [6]–[8], [17]–[19]. Compatibility describes the tenor of a social relationship, reflecting the shared history of social interactions exchanged within a dyad, as well as the similarities in the temperament of the two social partners toward each other. Finally, security describes the consistency of a social relationship over time. Despite the importance of social relationships to individual and group activities (see above), only a few studies have empirically tested the three component framework proposed by Cords and Aureli [9]: three studies on primates (chimpanzees, *Pan troglodytes*: [20]; Japanese macaques, *Macaca fuscata*: [21], spider monkeys, *Ateles geoffroyi*: [22]) and one on ravens *Corvus corax*, [23]). With the exception of the spider monkeys [22], through the use of principal component analyses (PCA), these studies showed that a series of behavioural variables measuring relationship quality (e.g. affiliative and agonistic behaviours), cluster according to the three component structure proposed by Cords and Aureli [9].

Here we examine whether relationship quality in wild Barbary macaques (*Macaca sylvanus*) can be described by the three components proposed by Cords and Aureli [9]. The Barbary macaque differs in the social system and/or the dominance style from the species that have so far been investigated on this topic [20]–[23]. Whereas macaques live in multi-male – multi-female groups [24], chimpanzees and spider monkeys live in fission-fusion societies [25], and ravens live in either non-breeder flocks or pair-bonds [23]. Moreover, Barbary macaques are considered to be characterised by a more egalitarian social system when compared to Japanese macaques [26]. Therefore, analysing if the three component structure describes relationship quality in our study species can tell us whether this framework applies to species with a different socio-ecology and phylogenetic history.

Two additional features seem to characterise social relationships in a range of group-living species. First, the distribution of social relationships within a group can be affected by a series of demographic and/or life-history parameters. High quality relationships are more frequently observed between individuals of similar age and/or sex, between relatives, or between individuals who have spent more time together in the same social group [14], [27]. For example, in sex-biased phylopatric species friendly relationships are expected to occur more frequently between individuals of the phylopatric sex [1], [24]. Barbary macaques live in multi-male – multi-female groups, characterised by female philopatry and male dispersal [24]. Therefore, we predicted that high quality relationships will be more frequent between individuals of the phylopatric sex (i.e. females), of the same age, and between close-ranking individuals [27].

A second feature of social relationships is that they are expected to frequently be asymmetric [9], [28] in terms of the different frequency with which two social partners exchange the same and/or different behaviours [21]. Such asymmetry can be measured by looking at whether the two members of a dyad exchange the same frequency of a given behaviour (e.g. the number of approaches given and received by the two members of a dyad is expected to be approximately equal in symmetric relationships). Asymmetry can result from both the trading of different social commodities (e.g. grooming for tolerance: [29], [30]) and the effect that differences in an individual's resource-holding potential (RHP; in terms of fighting ability, [31]) have on such trading. For example, dominant individuals can coerce grooming [32], [33] or mating opportunities [34] from subordinates, and receive more grooming from subordinates than vice-versa [35], [36]. The final aim of our study was thus to determine whether asymmetry characterises social relationships in the Barbary macaque. Barbary macaques are a relatively egalitarian species [26] which may result in a greater symmetry in the direction of social interactions within a dyad, in comparison to more despotic macaque species (e.g. *M. fuscata*, [21]). However, due to the importance of RHP and the trading of social commodities in shaping social relationships, we predicted that social relationships would be asymmetric in the Barbary macaque, similarly to what has been found in Japanese macaques [21].

## Methods

### a) Ethics statement

This study complies with Moroccan and UK regulations regarding the ethical treatment of research subjects. Research permission to conduct the study was granted by the Ethics Committee of the University of Lincoln, UK, and by the Haut Commissariat des Eaux et Forêts, Morocco (no permission IDs were given). This study was fully observational and our data collection did not affect the monkeys' welfare.

### b) Study subjects

Data were collected from two groups (‘Flat-face’ and ‘Large’) of wild Barbary macaques, living in a deciduous cedar and oak forest in the Middle-Atlas Mountains of Morocco (33°24′N–005°12′W). The study subjects relied on a completely natural diet. At the beginning of the study, the ‘Flat-face’ group consisted of 29 individuals (10 adult males, 1 sub-adult male, 8 adult females, 5 juveniles and 5 infants) and the ‘Large’ group consisted of 39 individuals (16 adult males, 3 sub-adult males, 10 adult females, 7 juveniles and 3 infants). We defined adults as being ≥5 years old, sub-adults 4–5 years, and juveniles 2–3 years [37], [38].

### c) Data collection

RM was responsible for the data collection with the help of four research assistants. Data were collected daily between 06.00 and 19.00 hours from June 2008 to September 2009 from all adult and sub-adult group members. Data were only collected when inter-observer reliability was above 95%. For all group member dyads (N = 577) the age combination of the dyad (adult-adult, subadult-subadult or adult-subadult), their sex combination (male-male, female-female or male-female), and their rank distance were recorded.

Scan sampling and focal sampling techniques [39] were used to collect data on the frequency and duration of social interactions for each dyad. In total, 792 scan samples and 1,102 hours of focal observations (mean hours/monkey ± SE = 18.71±2.10) were collected. Scan sampling data were collected hourly from all visible group members within ten minutes of the beginning of the scan. A single subject was never sampled more than once in a single scan. Data were collected on the activity of the study animals (i.e. resting, feeding, travelling, grooming or body contact), their ≤1.5 metre proximity to other study subjects, and on the identity of their social partners. Twenty minute continuous focal observations were used to collect data on close-proximity approaches (≤1.5 metre), grooming, grooming solicitations, aggression and agonistic support (see Table 1 for definitions). For each study monkey the order of focal observations was evenly distributed across the study period and time of day. A monkey was never sampled more than once in a single day. Scan and focal data were used to extract data on seven variables considered to represent the quality of a dyad's social relationship [21]. These behavioural variables were tolerance, proximity, grooming, grooming asymmetry, grooming solicitations, aggression, and agonistic support (Table 1). A major goal of this study was to compare the structure of social relationship qualities in wild Barbary macaques, with four other animal species (i.e. Japanese macaques, chimpanzees, spider monkeys and ravens). In order to make these comparisons it was crucial that a similar methodology was used (i.e. PCA: see below for details of this methodology), and that comparable variables were entered into this PCA. Therefore, the behavioural variables described in Table 1 were congruent with those used in previous studies on this topic [20]–[23].

**Table 1:** Behavioural measures of relationship quality.

Behaviour	Definition	Mean ± SEper dyad
Tolerance	Proportion of successful ≤1.5 metres approaches (approaches that were not followed by aggression or displacement for the first 30 seconds after the approach/all approaches) (%).	22.52±1.52
Proximity	Proportion of scans in ≤1.5 metre proximity (frequency/total number of scans) (%).	1.13±0.06
Grooming	Proportion of grooming exchanged (grooming given or received/total focal time) (%).	8.83±1.25
Grooming asymmetry	Grooming asymmetry index *	−0.04±0.05
Grooming solicitations	Frequency of grooming solicitation (i.e. when one monkey ‘presents’ a body part to be groomed by another monkey) (events/hour).	0.02±0.002
Aggression	Frequency of aggression exchanged (events/hour)	0.05±0.004
Agonistic support	Proportion of times in which one member of dyad supported another in an agonistic encounter (total support/total opportunity to support * †) (%).	0.003±0.001

*Based on a hypothetical dyad of individual A and B, the baseline asymmetry in the distribution of grooming was calculated using the following equation: (grooming received by individual A − grooming received by individual B)/(grooming received by individual A + grooming received by individual B).

† Based on a hypothetical dyad of individual A and B, the ‘opportunity to support’ was defined as the number of times individual A received aggression when individual B was in the group and potentially able to offer support to individual A.

### d) Data analysis

Following a similar methodology used in previous studies [20]–[23], we used PCA to explore the components of relationship quality in our study animals. PCA is a data reduction technique that organises numerous variables into a smaller number of composite variables called ‘principal components’. Principal components are described in terms of eigenvalues, component scores and factor loadings, and can be used to explain patterns of correlation within sets of multiple variables [40], [41]. It is common practice to name the principal components produced by a PCA in order to characterise the variables clustered within each component [20]–[23]. For example, following the theoretical framework of social relationship quality proposed by Cords and Aureli [9], social relationships in several animal species [20]–[23] have been described as having a component labelled ‘value’. These components were considered to measure the value of a social relationship because the variables clustered within it provide fitness benefits to social partners (e.g. grooming: [6]–[8], [17]–[19]).

Two PCAs were performed in the current study using the varimax rotation method and Kaiser normalisation [40]. The first PCA (PCA-1; run on the dyadic scores of the 7 variables; Table 1) was performed to analyse whether social relationships could be described by the three components of relationship quality proposed by Cords and Aureli [9]. Components were extracted with an eigenvalue >1 and variables were considered to have high loadings if they had a value of ≥0.5 or ≤−0.5 [21], [40]. Using the factor scores of each component obtained from PCA-1, generalised linear mixed models (GLMMs; [42]) were used to analyse the effect of sex combination (female-female, male-female and male-male), age combination (adult-adult vs. adult-subadult), and rank distance on each of the components of relationship quality. In all GLMMs the identity of the two members of a dyad were entered as two random factors. Group ID (‘Flat-face’ or ‘Large’ group) was entered as a ‘control’ fixed factor. Results for the control fixed factors are not shown here for the sake of brevity but can be found in the electronic appendix.

In order to explore the asymmetry of the social relationship, a second PCA (PCA-2) was performed on individual scores for each individual within a dyad. If social relationships were symmetrical, one would expect the giving and receiving of each behavioural variable to cluster in the same component [21]. It was not possible to calculate individual scores for the variables proximity and grooming asymmetry. As such, these two variables were excluded from PCA-2. The two PCAs were run using PASW Statistics v17 while GLMMs were performed using STATA v10.1 Software [43]. Social network graphs were built using Netdraw in UCINET 6.0 [44].

## Results

### a) Components of relationship quality

The first PCA (PCA-1) was performed on the seven behavioural variables considered to represent relationship quality using scores per dyad. PCA-1 produced three components explaining a combined variance of 61.41% (Table 2). Component 1 had positive loadings for grooming, grooming solicitations and proximity, explaining 30.78% of the variance. Following a similar rationale to previous authors [20], [21] this component was tentatively labelled ‘value’, as it was composed of behaviours that are beneficial for the fitness of the social partners [6]–[8], [17]–[19]. Component 2 had positive loadings for agonistic support and tolerance, and negative loadings for aggression, explaining 16.32% of the variance. This component was composed of behaviours requiring high levels of tolerance and low rates of aggression, and it was therefore tentatively labelled ‘compatibility’. Finally, component 3 (explaining 14.31%) had a high positive loading for grooming asymmetry, which is considered to measure the consistency or variability of a social relationship [20]–[21]. Thus, this component was labelled ‘security’. Note here that based on the formula used to calculate ‘grooming asymmetry’ (see Table 1 for details) high values for this variable indicate a more asymmetric distribution of grooming between two social partners.

**Table 2:** Varimax rotated component matrix of the principal component analysis run on the seven variables measuring relationship quality (using scores per dyad).

	Component		
	1	2	3
	(Value)	(Compatibility)	(Security)
Grooming	**.884**	.135	−.007
Proximity	**.752**	−.108	.146
Grooming solicitations	**.733**	−.130	−.172
Agonistic support	.017	**.721**	.069
Tolerance	.459	**.565**	−.082
Aggression	.205	**−.500**	.001
Grooming asymmetry	−.015	.031	**.980**
*% variance explained*	*30.55*	*16.24*	*14.62*

Variables with high loadings (i.e. ≥0.5 or ≤−0.5; [40]) are in bold.

**Figure 1:**
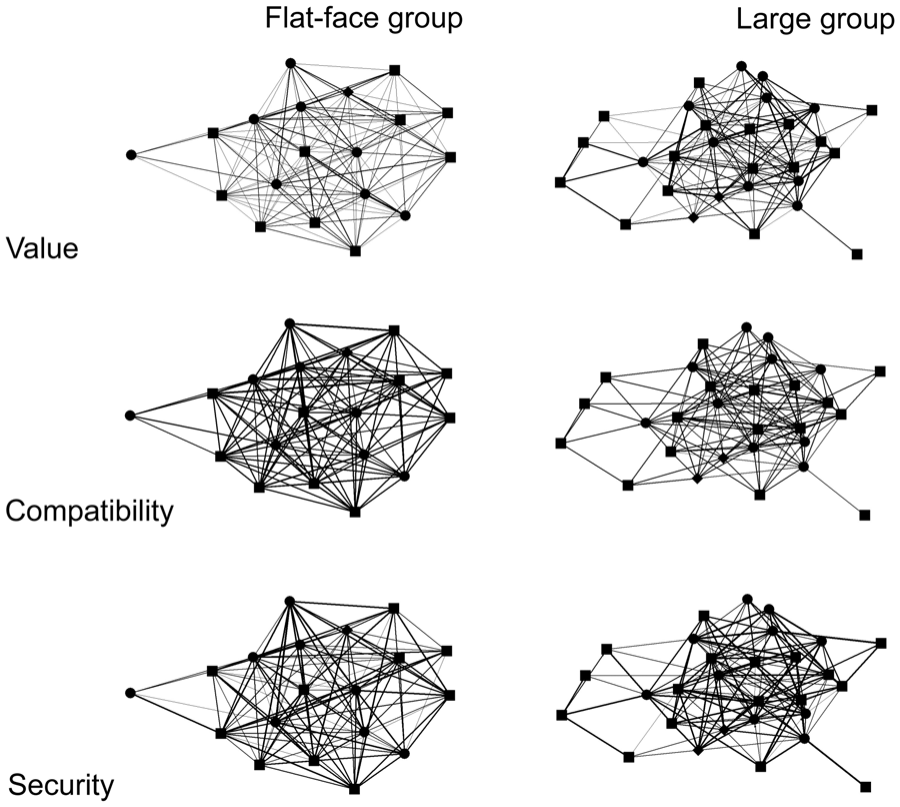
Social network graphs of the two study groups. Nodes represent individual group members (circles = adult females, squares = adult males, diamonds = sub-adult males). The thickness of the inter-connecting lines represents the tie-strength of principal component scores (i.e. value, compatibility and security) shared between dyads.

Six social networks are represented in Figure 1 to illustrate the distribution of social relationship qualities (i.e. principal component scores) within each social group.

### b) Predictors of relationship quality: sex, age and rank

**Figure 2:**
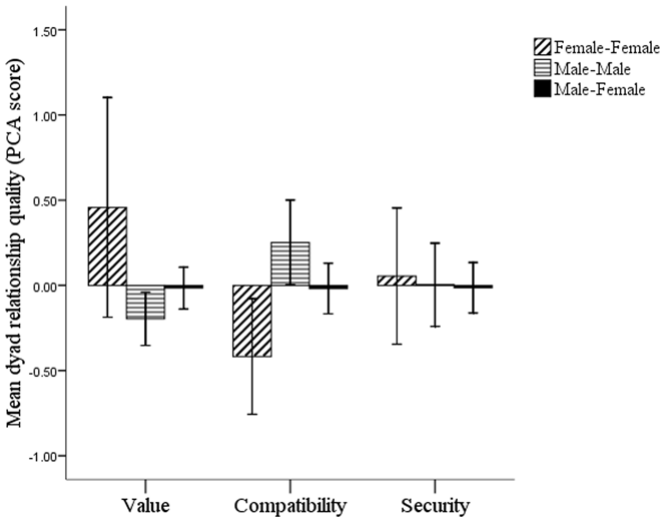
Histogram showing the mean relationship quality (PCA component scores) of female-female, male-male and female-male dyads.

Social relationships shared by female-female dyads were shown to be more ‘valuable’ than male-male and male-female dyads (Fig. 2, Table S1 and Table S2). However, social relationships shared by male-male and male-female dyads were shown to be more ‘compatible’ than female-female dyads (Fig. 2, Table S3 and Table S4). There was no significant difference across different sex combination dyads in how ‘secure’ their social relationship was (Fig. 2, Table S5 and Table S6).

Adult-adult dyads shared more ‘compatible’ relationships than adult-subadult dyads (adult-adult mean ± SE = 0.01±0.07, adult-subadult mean ± SE = −0.12±0.14; β ± SE = −0.56±0.22, 95% CIs = −0.98–−0.13, z = −2.58, N = 266, p<0.05; Table S7). When comparing adult-adult and adult-subadult relationship dyads there was no significant difference in how ‘valuable’ (adult-adult mean ± SE = 0.02±0.07, adult-subadult mean ± SE = −0.21±0.12; β ± SE = −0.15±0.22, 95% CIs = −0.58–0.29, z = −0.66, N = 266, p = 0.51; Table S8) or ‘secure’ (adult-adult mean ± SE = 0.00±0.06, adult-subadult mean ± SE = −0.02±0.20; β ± SE = 0.11±0.33, 95% CIs = −0.54–0.75, z = 0.32, N = 266, p = 0.75; Table S9) their social relationships were.

The quality of a dyad's social relationship was not predicted by the rank distance between dyad individuals in any of the three components of relationship quality (value: β ± SE = 0.01±0.01, 95% CIs = −0.01–0.03, z = 1.15, N = 266, p = 0.25; Table S8, compatibility: β ± SE = −0.01±0.01, 95% CIs = −0.02–0.01, z = −0.61, N = 266, p = 0.54; Table S7, security: β ± SE = 0.01±0.01, 95% CIs = −0.01–0.03, z = 1.02, N = 266, p = 0.31; Table S9).

### c) Asymmetry in relationship quality

A second PCA (PCA-2) was performed by entering the ten ‘given’ or ‘received’ parameters of the five behavioural variables for which the individual contribution within a dyad could be calculated. An asymmetric clustering of the giving and receiving parameter of the same behavioural variable was considered to reflect asymmetry in the distribution of social services within a relationship dyad [21]. The PCA produced four components explaining a combined variance of 57.96% (Table 3). With the exception of approaches, high loadings for the giving and receiving parameters of the same behavioural variable failed to cluster within the same component. As such, the two members of a dyad gave and received different rates of the same behaviour.

**Table 3:** Varimax rotated component matrix of the principal component analysis run on the ten variables measuring relationship quality (using scores per individual).

	Component			
	1	2	3	4
Grooming given	.354	.348	.425	.161
Grooming received	**.841**	.215	.074	.079
Aggression given	**.561**	−.155	−.016	−.150
Aggression received	−.217	**.652**	−.172	−.049
Grooming solicitations given	**.724**	.303	−.013	.038
Grooming solicitations received	.220	**.519**	.144	.124
Agonistic support given	−.042	−.044	−.022	**.960**
Agonistic support received	−.101	−.068	**.887**	−.047
Approach given	.164	**.736**	−.016	−.038
Approach received	.210	**.519**	.341	−.107
*% variance explained*	*18.49*	*17.96*	*11.40*	*10.12*

Variables with high loadings (i.e. ≥0.5 or ≤−0.5; [40]) are in bold.

## Discussion

Our findings, together with other published studies on this topic [20], [21], [23], indicate that a three component structure describes social relationships in distantly related animals (i.e. birds and primates). Moreover, this three component structure is found in species with different social systems (i.e. fission-fusion societies of chimpanzees, the multi-male – multi-female groups of macaques, and non-breeder flocks or pair-bonds in ravens). Therefore, Cords and Aureli's [9] predictions holds true even if animals of different species differ in their frequency or opportunity for social interactions with their group companions, and/or use species-specific behaviours (e.g. begging in chimpanzees, [20]) to establish and maintain social relationships.

Similarities and differences can be found within similarly named components across species. For example, congruent with the findings of the current study, grooming/preening is also found in the ‘value’ component of relationship quality in chimpanzees [20], Japanese macaques [21] and ravens [23]. Alternatively, aggression has been similarly found in the ‘compatibility’ component of relationship quality for chimpanzees and ravens, but it is considered to represent ‘security’ in the Japanese macaque. Making direct comparisons between studies needs to be done so with caution. Differences in the behavioural components constituting each component are dependent on a range of factors, including, for example, taxa/species-specific behaviours and differences in the social significance of similar behaviours observed across species. Despite such differences, a comparison of our findings with those of previous studies on this topic indicate that the three components proposed by Cords and Aureli [9] can describe relationship quality in a range of animal societies.

### a) Distribution of high quality relationships

#### Sex combination

Our study shows that in Barbary macaques, female-female dyads share more valuable relationships than female-male and male-male dyads. These results are congruent with the suggestion that in macaque societies, relationships among females hold more value than other group member dyads [24]. Moreover, these results support the view that individuals of the philopatric sex (i.e. females in the Barbary macaque) share higher quality relationships than individuals of the dispersing sex [14], probably due to the longer opportunities the former have to establish social bonds.

Majolo et al. [21] similarly found that sex combination was a predictor of relationship value in their study of Japanese macaques. However, in their study female-female dyads held more value than male-female dyads, but of similar value to male-male dyads. These results, as well as those of the current study highlight the importance of female philopatry in predicting relationship quality in macaques. Moreover, the difference in the relative quality of male-male social relationships between Barbary and Japanese macaques is indicative of the flexibility of macaque societies and their social organisation [24]. Female-female relationships were shown to be less compatible than other sex combination dyads. This result is puzzling considering that females had a higher value to their social relationships than males or hetero-sexual dyads. However, in studies of the social relationships shared by females, individuals that exchange high rates of grooming also exchange high rates of aggression [45]–[47]. This is in line with the results of our first PCA; female-female dyads were characterised by high rates of affiliative exchanges (i.e. a strong value component) and high rates of aggression (i.e. a weak compatibility component).

#### Age combination

There is evidence both in favour (chimpanzees, [20]) and against (Japanese macaques, [21]) the suggestion that similar-age social partners share higher quality relationships. In the current study, adult-adult dyads were more compatible than adult-subadult dyads. However, adult-adult dyads were not significantly more valuable or secure than adult-subadult dyads. de Waal and Luttrell [27] suggested that members of the same age cohort share similar needs in terms of resource access and also possess similar social power. Based on this assumption, these authors proposed the ‘similarity principle’ which states that individuals of a similar age are likely to be the best social partners to provide and exchange fitness benefits, and thus share more valuable relationships than different aged social partners. For example, in baboons (*Papio spp.*), grooming is more frequently exchanged between non-related individuals of a similar age [14]. Based on the suggestion that ‘compatibility’ reflects the shared history of social interactions between individuals [9], the findings of the current study confirm that the expected longevity of a relationship shared between adults is superior to that shared between an adult and a subadult, which positively contributes to their compatibility as social partners.

#### Rank distance

In the current study the rank distance between con-specifics did not predict the quality of their social relationship in any of the three dimensions of relationship quality. These results mirror those previously found in a study of Japanese macaques [21]. Similarly to the predictions of the ‘similarity principle’ [27], one might expect close-ranking social partners, to possess more valuable relationships than distant ranking social partners. However, dominant social partners are generally considered more valuable than subordinates in terms of the tolerance and agonistic support they have to offer [48], and dominant group members subsequently tend to have a larger social network than subordinates do [35], [49], [50]. Therefore, the value of forming relationships with social partners that share similar needs in term of resources and power as predicted by the ‘similarity principle’ [27] is likely to be counter-balanced by the superior resources available from maintaining relationships with high ranking individuals (i.e. large rank distances between members of the same dyad). This could ultimately ‘level-out’ differences in relationship quality between dyads of different rank distance, explaining the absence of a rank-distance effect on the distribution of relationship quality in our study groups.

### b) Relationship asymmetry

In contrast to the PCA-1 (run on dyadic scores), four components of relationship quality were identified by PCA-2 (run on individual scores). These results reflect those found in a previous study of Japanese macaques [21] and suggest that the three component structure of social relationship quality proposed by Cords and Aureli [9] does not take into account asymmetries in social relationships. Differences in RHP [31] and dominance are considered to play a major role in controlling the asymmetric distribution of resources between social partners [9], [14]. Moreover, the exchange of resources between social partners does not always involve the direct exchange of the same resource [51]. For example, grooming has been observed to be either directly reciprocated or exchanged for a range of other social resources [29], [30], [52], [53]. The asymmetric clustering of the giving and receiving parameters of the same behavioural variable in the current study, supports the notion that social commodities are exchanged and interchanged and are not always directly reciprocated [51].

In component one of PCA-2, grooming received clustered with aggression given, indicating that social partners that exchanged the most aggression, also exchanged the most grooming. This finding supports our explanation for the low compatibility of female-female social relationships found in our study (see above). Tentatively these results may also suggest that aggression, or the threat of aggression, is used to control the input a subordinate social partner makes to their relationship (i.e. coercion and/or punishment, [32], [54]). This would support the claim that the apparent threat of aggression causes subordinate individuals to preferentially groom those that aggress them the most (i.e. in an attempt to appease their aggression; [45]–[47]), and that more dominant individuals (i.e. individuals that aggress others more often) tend to receive more grooming than subordinates [35], [36]. Alternatively, the results may be an artefact of the likelihood that highly affiliative social partners are inherently likely to exchange more aggression as they spend more time together. It is important to note that these findings are not contrary to the ‘no rank effect’ finding from PCA-1 (see above). PCA-2 describes the directional distribution of grooming and aggression. Conversely, PCA-1 does not describe the directionality of grooming, but instead describes the total grooming exchanged within a dyad. Although rank differences may affect the directional distribution of grooming within a dyad (i.e. the results of PCA-2), it does not necessarily affect the total grooming exchanged (i.e. the results of PCA-1). Therefore, the absence of a rank related effect in PCA-1 is not contrary to the findings and implications of PCA-2 made here.

Differences in social power and dominance are likely to affect the relative value an individual poses on their social relationship [9], [27]. However, social bonding is often described by direct reciprocity [55] and in studies in which the quality of a social relationship is considered to affect the function of specific social behaviours, relationship quality is most commonly described in terms of a dyad's *shared* relationship quality. For example, the occurrence of reconciliation in animal societies has been shown to be predicted by the shared quality of a dyad's social relationship [11], [56]–[58]. The results of the current study highlight the importance of considering asymmetry when exploring the dimensions of quality within a social relationship and may also explain the low observed frequencies of reciprocity in animal societies. Therefore, asymmetry as well as reciprocity should be considered when describing the social bonds shared by con-specifics [55].

## Supporting Information

Table S1 GLMM results for the relationship between social relationship ‘value’ and dyad sex (FF vs. MM).
(DOC)

Table S2 GLMM results for the relationship between social relationship ‘value’ and dyad sex (FF vs. MF).
(DOC)

Table S3 GLMM results for the relationship between social relationship ‘compatibility’ and dyad sex (FF vs. MM).
(DOC)

Table S4 GLMM results for the relationship between social relationship ‘compatibility’ and dyad sex (FF vs. MF).
(DOC)

Table S5 GLMM results for the relationship between social relationship ‘security’ and dyad sex (FF vs. MM).
(DOC)

Table S6 GLMM results for the relationship between social relationship ‘security’ and dyad sex (FF vs. MF).
(DOC)

Table S7 GLMM results for the relationship between social relationship ‘compatibility’, dyad age combination and rank difference.
(DOC)

Table S8 GLMM results for the relationship between social relationship ‘value’, dyad age combination and rank difference.
(DOC)

Table S9 GLMM results for the relationship between social relationship ‘security’, dyad age combination and rank difference.
(DOC)
