# Supplementary material for: Exploring the Components, Asymmetry and Distribution of Relationship Quality in Wild Barbary Macaques (Macaca sylvanus)
Source: PLoS One. 2011 Dec 14;6(12):e28826. doi: 10.1371/journal.pone.0028826 (PMC3237547; doi:10.1371/journal.pone.0028826)
Supplement: Table S1 — GLMM results for the relationship between social relationship ‘value’ and dyad sex (FF vs. MM). (DOC) [file pone.0028826.s001.doc]

###### Table S1. GLMM results for the relationship between social relationship ‘value’ and dyad sex (FF vs. MM)

|  | **β ± SE** | **Z** | **P** | **N** | **95% CIs** |
| --- | --- | --- | --- | --- | --- |
| Group | -1.05 ± 0.27 | -3.86 | <0.001 | 107 | -1.6 – -0.52 |
| Rank difference | 0.26 ± 0.03 | 0.72 | 0.47 | 107 | -0.04 – 0.09 |
| Age combination | -0.20 ± 0.43 | -0.47 | 0.64 | 107 | -1.04 – 0.63 |
| FF vs. MM | 0.60 ± 0.29 | 2.10 | 0.04 | 107 | 0.04 – -1.16 |
