# Supplementary material for: Exploring the Components, Asymmetry and Distribution of Relationship Quality in Wild Barbary Macaques (Macaca sylvanus)
Source: PLoS One. 2011 Dec 14;6(12):e28826. doi: 10.1371/journal.pone.0028826 (PMC3237547; doi:10.1371/journal.pone.0028826)
Supplement: Table S2 — GLMM results for the relationship between social relationship ‘value’ and dyad sex (FF vs. MF). (DOC) [file pone.0028826.s002.doc]

Table S2. GLMM results for the relationship between social relationship ‘value’ and dyad sex (FF vs. MF)

|  | **β ± SE** | **Z** | **P** | **N** | **95% CIs** |
| --- | --- | --- | --- | --- | --- |
| Group | -0.98 ± 0.19 | -5.10 | <0.001 | 195 | -1.36 – -0.61 |
| Rank difference | 0.02 ± 0.02 | 1.22 | 0.22 | 195 | -0.01 – 0.05 |
| Age combination | -0.27 ± 0.46 | -0.60 | 0.55 | 195 | -1.12 – 0.62 |
| FF vs. MF | -0.51 ± 0.24 | -2.08 | 0.04 | 195 | -0.99 – 0.03 |
