# Supplementary material for: Exploring the Components, Asymmetry and Distribution of Relationship Quality in Wild Barbary Macaques (Macaca sylvanus)
Source: PLoS One. 2011 Dec 14;6(12):e28826. doi: 10.1371/journal.pone.0028826 (PMC3237547; doi:10.1371/journal.pone.0028826)
Supplement: Table S3 — GLMM results for the relationship between social relationship ‘compatibility’ and dyad sex (FF vs. MM). (DOC) [file pone.0028826.s003.doc]

Table S3. GLMM results for the relationship between social relationship ‘compatibility’ and dyad sex (FF vs. MM)

|  | **β ± SE** | **Z** | **P** | **N** | **95% CIs** |
| --- | --- | --- | --- | --- | --- |
| Group | -0.82 ± 0.24 | -3.46 | 0.001 | 107 | -1.23 – -0.36 |
| Rank difference | -0.05 ± 0.03 | -2.06 | 0.04 | 107 | -0.10 – -0.00 |
| Age combination | -0.22 ± 0.34 | -0.66 | 0.51 | 107 | -0.89 – 0.44 |
| FF vs. MM | -0.98 ± 0.25 | -3.92 | <0.001 | 107 | -1.47 - -0.49 |
