# Supplementary material for: Exploring the Components, Asymmetry and Distribution of Relationship Quality in Wild Barbary Macaques (Macaca sylvanus)
Source: PLoS One. 2011 Dec 14;6(12):e28826. doi: 10.1371/journal.pone.0028826 (PMC3237547; doi:10.1371/journal.pone.0028826)
Supplement: Table S4 — GLMM results for the relationship between social relationship ‘compatibility’ and dyad sex (FF vs. MF). (DOC) [file pone.0028826.s004.doc]

Table S4. GLMM results for the relationship between social relationship ‘compatibility’ and dyad sex (FF vs. MF)

|  | **β ± SE** | **Z** | **P** | **N** | **95% CIs** |
| --- | --- | --- | --- | --- | --- |
| Group | -0.92 ± 0.18 | -5.20 | <0.001 | 195 | -1.27 – -0.57 |
| Rank difference | -0.02 ± 0.01 | -1.36 | 0.17 | 195 | -0.05 – 0.01 |
| Age combination | -0.58 ± 0.38 | -1.53 | 0.13 | 195 | -1.33 – 0.16 |
| FF vs. MF | 0.73 ± 0.20 | 3.56 | <0.001 | 195 | 0.33 – 1.13 |
