# Supplementary material for: Exploring the Components, Asymmetry and Distribution of Relationship Quality in Wild Barbary Macaques (Macaca sylvanus)
Source: PLoS One. 2011 Dec 14;6(12):e28826. doi: 10.1371/journal.pone.0028826 (PMC3237547; doi:10.1371/journal.pone.0028826)
Supplement: Table S5 — GLMM results for the relationship between social relationship ‘security’ and dyad sex (FF vs. MM). (DOC) [file pone.0028826.s005.doc]

Table S5. GLMM results for the relationship between social relationship ‘security’ and dyad sex (FF vs. MM)

|  | **β ± SE** | **Z** | **P** | **N** | **95% CIs** |
| --- | --- | --- | --- | --- | --- |
| Group | 0.07 ± 0.30 | 0.24 | 0.81 | 107 | -0.52 – 0.66 |
| Rank difference | 0.06 ± 0.03 | 2.10 | 0.04 | 107 | 0.00 – 0.12 |
| Age combination | -0.34 ± 0.45 | -0.76 | 0.45 | 107 | -1.23 – 0.54 |
| FF vs. MM | 0.23 ± 0.31 | 0.74 | 0.46 | 107 | -0.38 – 0.85 |
