# Supplementary material for: Exploring the Components, Asymmetry and Distribution of Relationship Quality in Wild Barbary Macaques (Macaca sylvanus)
Source: PLoS One. 2011 Dec 14;6(12):e28826. doi: 10.1371/journal.pone.0028826 (PMC3237547; doi:10.1371/journal.pone.0028826)
Supplement: Table S6 — GLMM results for the relationship between social relationship ‘security’ and dyad sex (FF vs. MF). (DOC) [file pone.0028826.s006.doc]

Table S6. GLMM results for the relationship between social relationship ‘security’ and dyad sex (FF vs. MF)

|  | **β ± SE** | **Z** | **P** | **N** | **95% CIs** |
| --- | --- | --- | --- | --- | --- |
| Group | 0.05 ± 0.26 | 0.20 | 0.84 | 195 | -0.46 – 0.57 |
| Rank difference | 0.01 ± 0.02 | 0.67 | 0.50 | 195 | -0.02 – 0.05 |
| Age combination | 0.19 ± 0.48 | 0.40 | 0.69 | 195 | -0.75 – 1.13 |
| FF vs. MF | -0.29 ± 0.25 | -1.16 | 0.24 | 195 | -0.79 – 0.20 |
