# Supplementary material for: Exploring the Components, Asymmetry and Distribution of Relationship Quality in Wild Barbary Macaques (Macaca sylvanus)
Source: PLoS One. 2011 Dec 14;6(12):e28826. doi: 10.1371/journal.pone.0028826 (PMC3237547; doi:10.1371/journal.pone.0028826)
Supplement: Table S7 — GLMM results for the relationship between social relationship ‘compatibility’, dyad age combination and rank difference. (DOC) [file pone.0028826.s007.doc]

Table S7. GLMM results for the relationship between social relationship ‘compatibility’, dyad age combination and rank difference

|  | **β ± SE** | **Z** | **P** | **N** | **95% CIs** |
| --- | --- | --- | --- | --- | --- |
| Group | -1.01 ± 0.16 | -6.51 | <0.001 | 266 | -1.32 – -0.71 |
| Sex combination | -0.41 ± 0.10 | -4.14 | <0.001 | 266 | -0.60 – -0.22 |
| Rank difference | -0.01 ± 0.01 | -0.61 | 0.54 | 266 | -0.02 – 0.01 |
| Age combination | -0.56 ± 0.22 | -2.58 | 0.01 | 266 | -0.98 – -0.13 |
