# Supplementary material for: Exploring the Components, Asymmetry and Distribution of Relationship Quality in Wild Barbary Macaques (Macaca sylvanus)
Source: PLoS One. 2011 Dec 14;6(12):e28826. doi: 10.1371/journal.pone.0028826 (PMC3237547; doi:10.1371/journal.pone.0028826)
Supplement: Table S8 — GLMM results for the relationship between social relationship ‘value’, dyad age combination and rank difference. (DOC) [file pone.0028826.s008.doc]

Table S8. GLMM results for the relationship between social relationship ‘value’, dyad age combination and rank difference

|  | **β ± SE** | **Z** | **P** | **N** | **95% CIs** |
| --- | --- | --- | --- | --- | --- |
| Group | -0.89 ± 0.14 | -6.25 | <0.001 | 266 | -1.17 – -0.61 |
| Sex combination | 0.26 ± 0.10 | 2.61 | 0.01 | 266 | 0.06 – 0.46 |
| Rank difference | 0.01 ± 0.01 | 1.15 | 0.25 | 266 | -0.01 – 0.03 |
| Age combination | -0.15 ± 0.22 | -0.66 | 0.51 | 266 | -0.58 – 0.29 |
