# Supplementary material for: Exploring the Components, Asymmetry and Distribution of Relationship Quality in Wild Barbary Macaques (Macaca sylvanus)
Source: PLoS One. 2011 Dec 14;6(12):e28826. doi: 10.1371/journal.pone.0028826 (PMC3237547; doi:10.1371/journal.pone.0028826)
Supplement: Table S9 — GLMM results for the relationship between social relationship ‘security’, dyad age combination and rank difference. (DOC) [file pone.0028826.s009.doc]

Table S9. GLMM results for the relationship between social relationship ‘security’, dyad age combination and rank difference

|  | **β ± SE** | **Z** | **P** | **N** | **95% CIs** |
| --- | --- | --- | --- | --- | --- |
| Group | 0.13 ± 0.23 | 0.57 | 0.57 | 266 | -0.32 – 0.58 |
| Sex combination | 0.09 ± 0.13 | 0.68 | 0.50 | 266 | -0.17 – 0.35 |
| Rank difference | 0.01 ± 0.01 | 1.02 | 0.31 | 266 | -0.01 – 0.03 |
| Age combination | 0.11 ± 0.33 | 0.32 | 0.75 | 266 | -0.54 – 0.75 |
